# Cognitive impairment and C-reactive protein in clinically stable schizophrenia outpatients: a focus on sex differences

**DOI:** 10.1038/s41598-020-73043-x

**Published:** 2020-09-29

**Authors:** Francesco Dal Santo, Leticia González-Blanco, Leticia García-Álvarez, Lorena de la Fuente-Tomás, Ángela Velasco, Clara María Álvarez-Vázquez, Clara Martínez-Cao, Pilar A. Sáiz, María Paz García-Portilla, Julio Bobes

**Affiliations:** 1 Área de Psiquiatría, Universidad de Oviedo, Oviedo, Spain; 2 Servicio de Salud del Principado de Asturias (SESPA), Oviedo, Spain; 3 Centro de Investigación Biomédica en Red de Salud Mental (CIBERSAM), Oviedo, Spain; 4 Instituto de Investigación Sanitaria del Principado de Asturias (ISPA), Oviedo, Spain; 5 Instituto de Neurociencias del Principado de Asturias (INEUROPA), Oviedo, Spain; 6 Departamento de Psicología, Universidad de Oviedo, Oviedo, Spain; 7 Centro de Salud Mental La Corredoria, Alfredo Blanco s/n, Oviedo, Spain

**Keywords:** Neuroscience, Biomarkers

## Abstract

Although previous findings identified an association between C-reactive protein (CRP) levels, and impaired cognitive functions in patients with schizophrenia (SZ), little is currently known about the relationship between inflammation, cognition, and sex in SZ. The current study aimed to explore the association between peripheral inflammation and cognitive impairment in SZ as a function of sex. The sample included 132 clinically stable patients with SZ, of whom 82 were males (62.1%) and 50 females (37.9%). Sociodemographic data were collected, an accurate assessment was performed using the Positive and Negative Syndrome (PANSS), Clinical Assessment Interview for Negative Symptoms (CAINS), and Calgary Depression (CDS) scales, and the MATRICS Consensus Cognitive Battery (MCCB), and CRP levels were tested. A Pearson correlation and multiple regression analyses, including potential confounding factors, were performed. We found an inverse association between CRP levels and performance on visual learning (r = − 0.386, p = 0.006) domain in female patients only, whereas no correlations were found in males. The regression model for women retained age (β = − 0.319, p = 0.017), the CAINS-MAP score (β = − 0.247, p = 0.070), and the CRP (β = − 0.321, p = 0.013) as predictors of visual learning. Our results suggest the possible existence of sex-specific modulation of the association between systemic inflammation and the cognitive features of the illness.

## Introduction

The inflammatory hypothesis of schizophrenia (SZ) postulates the implication of inflammatory processes and neural-immune interactions in its pathogenesis of the illness and some of its neurobiological correlates^1^. The interactions between the immune system, systemic inflammation, and the brain are complex, and may account for changes in mood, cognition, and behaviour in a broad range of psychiatric disorders^2^. Moreover, symptoms of SZ have been described in several inflammatory diseases^3^. C-reactive protein (CRP) represents a reliable, easy, and inexpensive marker of peripheral inflammation^4^. Plasmatic CRP has been found to correlate with central immune activity in patients with psychiatric disorders^5^, and recent literature shows a growing interest in its potential role as a biomarker of schizophrenia^6^. Elevated CRP levels have been associated with more severe psychopathology and a wide range of impaired cognitive functions in SZ in both inpatient and outpatient samples^6^, as well as in other chronic psychiatric syndromes such as bipolar disorder (BD)^7^. Sex and hormonal status could play a role in determining this association, and in a community-dwelling elderly population, the relationship between CRP concentration and cognitive decline was observed only in women^8^.

Sex differences have been reported in various aspects of cognition across several species including humans, e.g. while males tend to outperform women in spatial skills, females perform better on verbal tasks^9^. Results in SZ are inconsistent: in some cases, typical sex differences are preserved while other studies have found that sex differences in cognitive function may be diminished or even reversed^10^. A whole range of other factors could potentially contribute to sex differences, and little is currently known about the relationship between inflammation, cognition, and sex in schizophrenia. Therefore, we hypothesised that the relation between CRP and cognition would be different in males and females.

This study aimed to explore the association between peripheral inflammation, psychopathological features, and cognitive impairment in SZ as a function of sex.

## Methods

### Design and population

A total of 150 outpatients with schizophrenia were consecutively recruited among those treated at community mental health facilities in Asturias (Spain), who were on stable maintenance treatment for at least 3 months. The diagnosis was made by a psychiatrist and confirmed with a structured clinical interview (SCID). All patients were Caucasian.

Acute or chronic inflammatory comorbidities (e.g. infection, allergies, cancer, and autoimmune disorders) and treatment with immunosuppressants or vaccines in the 6 months before enrolment or with anti-inflammatory drugs in the 2 days before blood collection were exclusion criteria.

This was a secondary analysis of data from 2 studies (PI13/02263 and PI16/01761), which aimed to identify specific biomarkers and to develop a staging model of the disease. All participants received information about the purposes and protocol of the study and signed the informed consent before any study procedures were performed. The study was approved by the Regional Clinical Research Ethics Committee (Comité de Ética de la Investigación del Principado de Asturias, Ref. 25/2014) and performed in accordance with the current guidelines and regulations.

### Measurements

#### Clinical assessment

An ad hoc protocol was employed for demographic and clinical characteristics. A comprehensive psychopathological assessment was done at baseline, including the Spanish versions of the Positive and Negative Syndrome Scale (PANSS)^11^, the Calgary Depression Scale (CDS)^12^, and the Clinical Assessment Interview for Negative Symptoms (CAINS), which is organized into two subscales measuring deficits in motivation and pleasure (CAINS-MAP) and expression (CAINS-EXP)^13^.

To evaluate the cognitive performance, we employed the Spanish version of the MATRICS Consensus Cognitive Battery (MCCB), which tests seven cognitive domains: processing speed, attention/vigilance, working memory, verbal learning, visual learning, reasoning and problem solving, and social cognition^14^.

#### Laboratory tests

Blood samples were obtained by venipuncture between 8:00 and 10:00 a.m. after a confirmed overnight fast. Quantitative analysis of CRP in plasma was performed by immunoturbidimetric assay (Roche/Hitachi Cobas c501). In keeping with previous studies^15^, an abnormal CRP level was defined as > 0.3 mg/dL and patients with CRP levels > 3.0 mg/dL, corresponding to acute inflammation, were not included in the final analyses.

### Statistical analysis

The statistical analysis was performed using IBM SPSS Statistics for Windows, version 24.0. The statistical significance was set at 0.05. Descriptive statistical analyses were performed using means, standard deviations (SD), and percentages. Categorical data were analysed using the chi-square test, and Student's t-test for independent samples was used for continuous data.

Pearson correlations were first applied to explore the possible relationship between CRP levels and cognitive domains. The Bonferroni correction was applied to correlation analysis to control for multiple testing on the domains of the MATRICS battery (n = 7), and we set the α = 0.05/7 = 0.007. Then, we conducted linear multiple regression analyses using CRP as the independent variable and the domains that significantly correlated with CRP as dependent variables. To control for the possible confounding effect of psychopathology, we performed a correlation analysis between the cognitive domains that correlated with CRP and the psychopathological scores, including those which significantly correlated as covariates in the regression model. Moreover, other potential confounding factors as age, body mass index and psychopharmacological treatment (use of benzodiazepines and chlorpromazine equivalents) were included based on expert criteria and the existing literature, for their possible influence on CRP or cognition. Separate analyses were performed for men and women.

## Results

Of the 150 patients enrolled, 18 (12.0%) were excluded for: (a) having a CRP > 3.0 mg/dL (n = 3, 2.0%), (b) missing CRP value (n = 11, 7.3%), or (c) incomplete cognitive evaluation (n = 4, 2.6%). Thus 132 patients were included in the final analysis. Demographic and clinical variables of the study sample are reported by sex in Table 1.

**Table 1:** Sociodemographic data, clinical assessment and biomarkers, according to sex.[(mean, SD) or (N, %)].

	Men (n = 82; 62.1%)	Women (n = 50; 37.9%)	Test (p)
Age	35.41 (11.52)	40.16 (12.35)	t = − 2.234 (0.027)
Illness duration (years)	8.38 (9.01)	9.84 (12.11)	t = − 0.787 (0.433)
Hospital admissions	1.66 (2.50)	1.40 (1.64)	t = 0.583 (0.561)
BMI (kg/m^2^)	28.62 (5.66)	27.60 (5.80)	t = 0.997 (0.321)
CPZeq (mg/day)	776.23 (551.69)	501.42 (539.41)	t = 2.799 (0.006)
BZD treatment, n (%)	43 (52.4%)	19 (38.0%)	Χ^2^ = 2.600 (0.107)
Smoking, n (%)	48 (58.5%)	17 (34.0%)	Χ^2^ = 7.482 (0.006)
**Clinical scales**			
PANSS positive	13.63 (5.34)	11.68 (5.24)	t = 2.053 (0.042)
PANSS negative	19.39 (5.70)	16.96 (5.71)	t = 0.993 (0.019)
PANSS GP	31.70 (8.00)	27.22 (7.90)	t = 0.642 (0.002)
CAINS MAP	20.27 (9.47)	18.16 (10.21)	t = 1.205 (0.230)
CAINS EXP	6.84 (4.10)	5.66 (4.50)	t = 1.548 (0.124)
CDS	3.23 (3.95)	3.00 (4.24)	t = 0.318 (0.751)
**MCCB (T-scores)**			
Speed of processing	33.39 (18.36)	32.26 (11.52)	t = 0.390 (0.697)
Attention/vigilance	34.55 (10.80)	34.62 (11.94)	t = − 0.035 (0.973)
Working memory	38.80 (14.37)	40.61 (12.55)	t = − 0.730 (0.467)
Verbal learning	38.84 (9.75)	41.96 (12.14)	t = − 1.622 (0.107)
Visual learning	35.72 (14.04)	38.27 (14.35)	t = − 0.996 (0.321)
Reasoning and problem solving	35.52 (9.61)	36.35 (7.35)	t = − 0.551 (0.583)
Social cognition	43.46 (16.15)	47.69 (20.21)	t = − 1.232 (0.222)
CRP (mg/dL)	0.33 (0.36)	0.37 (0.51)	t = − 0.463 (0.645)
CRP > 0.3 mg/dL, n (%)	22 (26.8%)	13 (26.0%)	Χ^2^ = 0.011 (0.917)

BMI: Body Mass Index, BZD: Benzodiazepines, CAINS: Clinical Assessment Interview for Negative Symptoms, -EXP: Expression subscale, -MAP: Motivation and Pleasure subscale, CDS: Calgary Depression Scale, CPZeq: Chlorpromazine equivalent, CRP: C-Reactive Protein; MCCB: MATRICS Consensus Cognitive Battery, PANSS: Positive and Negative Syndrome Scale, -GP: General Psychopathology.

The mean age was 37.21 ± 12.00 years, and most of the subjects were male (n = 82, 62.1%). The mean length of illness was 8.94 ± 10.29 years, with no sex differences even though women in the sample were older (40.16 ± 12.35 vs. 35.41 ± 11.52 years, p = 0.027).

Most of the patients were on treatment with one (n = 77, 58.3%) or two (n = 36, 27.3%) antipsychotics, being paliperidone and olanzapine the most prescribed drugs (Supplementary Table S1). At the time of the study, 9 patients (6.8%) were not taking any antipsychotic treatment, 122 patients (92.4%) were on treatment with at least one atypical antipsychotic, and only one male patient (0.8%) was on monotherapy with a typical antipsychotic. Males were treated with a higher dose of antipsychotic medication, measured as chlorpromazine equivalents (Table 1).

Both sexes had mean CRP values above the level defined as abnormal (> 0.3 mg/dL). No significant sex-related difference in CRP levels was found (men 0.33 ± 0.36 mg/dL vs women 0.37 ± 0.51, p > 0.05). Overall, 35 patients (26.5%) presented peripheral inflammation, with no sex difference observed. CRP showed a positive correlation with BMI in female subjects only (r = 0.481, p < 0.001), while this result was not observed in males (r = 0.174, p = 0.120). No differences in CRP levels were found between smokers and no-smokers, neither in males (t = 0.650, p = 0.518) nor in females (t = − 0.702, p = 0.486).

Significant differences between men and women were found in PANSS Positive (13.63 ± 5.34 vs. 11.68 ± 5.24, p = 0.042), Negative (19.39 ± 5.70 vs. 16.96 ± 5.71, p = 0.019), and General (31.70 ± 8.00 vs. 27.22 ± 7.90, p = 0.002) subscales. No differences were detected in either CAINS scores or depressive symptoms measured by CDS. None of the psychopathological variables significantly correlated with CRP levels (Table 2).

**Table 2:** Correlation between CRP level and psychopathological and cognitive variables [r (p)].

	Men (n = 82)	Women (n = 50)
**Clinical scales**		
PANSS positive	0.091 (0.418)	− 0.090 (0.536)
PANSS negative	− 0.179 (0.109)	0.089 (0.540)
PANSS GP	− 0.048 (0.669)	− 0.088 (0.545)
CAINS MAP	0.025 (0.821)	0.240 (0.094)
CAINS EXP	− 0.147 (0.188)	0.143 (0.321)
CDS	0.012 (0.915)	− 0.153 (0.288)
**MCCB (T-scores)**		
Speed of processing	− 0.097 (0.385)	− 0.248 (0.083)
Attention/vigilance	− 0.108 (0.339)	− 0.158 (0.274)
Working memory	− 0.161 (0.148)	− 0.050 (0.731)
Verbal learning	− 0.096 (0.389)	− 0.280 (0.049)
Visual learning	− 0.093 (0.408)	− 0.386 (0.006)
Reasoning and problem solving	− 0.050 (0.655)	− 0.201 (0.166)
Social cognition	0.041 (0.718)	− 0.074 (0.615)

CAINS: Clinical Assessment Interview for Negative Symptoms, -EXP: Expression subscale, -MAP: Motivation and Pleasure subscale, CDS: Calgary Depression Scale, CRP: C-Reactive Protein; MCCB: MATRICS Consensus Cognitive Battery, PANSS: Positive and Negative Syndrome Scale, -GP: General Psychopathology.

Scores (T-scores) on cognitive domains of the MCCB are reported in Table 1, with no sex differences observed. Of the analysed domains (Table 2) and with the significance set to α = 0.007, only the visual learning (r = − 0.386, p = 0.006) domain reached statistical significance and showed a negative correlation with CRP levels in females only. The inclusion of the three patients with CRP levels > 3.0 mg/dL (1 male and 2 females) did not change the results in males, but the association was no longer significant in females (p > 0.05). However, their CRP levels were extreme outliers. For this reason, and in accordance with previous literature^15,24^, patients with CRP levels > 3.0 mg/dL were excluded from the analyses.

The correlation analysis between visual learning domain, and psychopathological features showed a negative correlation between visual learning score and PANSS Negative (males: r = − 0.488, p < 0.001; females: r = − 0.376, p = 0.008) and General (males: r = − 0.346, p < 0.001; females: r = − 0.320, p = 0.025) subscales, the CAINS-MAP (males: r = − 0.597, p < 0.001; females: r = − 0.448, p < 0.001), and the CAINS-EXP (males: r = − 0.539, p < 0.001; females: r = − 0.340, p = 0.017) (Supplementary Table S2). These variables, along with CRP, age, body mass index, and psychopharmacological treatment (use of benzodiazepines and chlorpromazine equivalents) were included in the regression analysis for both males and females.

The multiple regression model for visual learning included in males (R^2^ = 0.453, F = 32.312, p < 0.001) the CAINS-MAP score (β = − 0.560, p < 0.001) and the chlorpromazine equivalent dose (β = − 0.296, p = 0.001), while in females (R^2^ = 0.365, F = 8.610, p < 0.001) it retained the age (β = − 0.319, p = 0.017), the CAINS-MAP score (β = − 0.247, p = 0.070), and the CRP (β = − 0.321, p = 0.013).

## Discussion

The main finding of the current study was an inverse association between systemic inflammation, measured by serum CRP levels, and performance on the visual learning subdomain in female patients with SZ only, suggesting the possible existence of sex-specific differences underlying the cognitive features of the illness, as we hypothesised. This effect was obtained even after controlling for covariates that could be associated with CRP levels or cognitive functioning. This association was no longer significant when three extreme outliers with CRP levels > 3.0 mg/dL (1 male and 2 females) were included. However, it should be mentioned that one of the two females with CRP levels > 3.0 mg/dL presented a performance above the 80th percentile on the visual learning task, which may have influenced this discrepancy. To our knowledge, this is the first study to analyse the differential association of CRP with cognition in patients with SZ as a function of sex.

Impairments in sensory processing are frequently presented in patients with schizophrenia, including visual perception and processing^16^. In recent years, several studies tried to elucidate the mechanisms underlying cognitive impairment in SZ, with a number of studies testing the association between CRP levels and cognitive performance in SZ patients^6^. In all studies that also included healthy controls, CRP levels were significantly higher in the patient group^6^. CRP levels were associated with cognitive impairment in both inpatient^17,18^ and outpatient^15,19^ samples. Hence, the findings of our research are consistent with the existing literature, which shows an inverse correlation between CRP and a wide range of cognitive functions^15^, including short-term memory^17^, delayed memory^18^, attention^18,19^, and verbal and visual episodic learning abilities^15^. A longitudinal study observed that a reduction in CRP level significantly increased verbal abilities in the period from 1 to 6 months after hospital discharge^19^.

Furthermore, our findings are of interest in light of studies indicating an association between CRP levels and cognitive impairment in other psychiatric diseases such as BD^20,21^, suggesting that the activation of inflammatory processes can affect cognition in a transdiagnostic context^7^. Interestingly, a recent study found a correlation between CRP levels and verbal learning abilities in females with BD but not in males^22^.

Biological differences between males and females are found at multiple levels, and the existing literature shows a paucity of research directly examining sex differences, disregarding the unique presentation of a broad range of diseases in each sex^9^. In our sample, we found a sex difference by age without differences in the duration of illness, which may be due to the earlier age at onset in men than in women^10^. Men of our sample also had a more severe clinical presentation according to the PANSS, which is consistent with the worse short and medium-term outcomes associated with male sex^23^. As with the cognitive dimension of SZ, we did not find sex differences in the MATRICS subdomains, while the existing literature shows mixed results^10^. In keeping with previous studies^15^, our CRP levels did not differ between sexes, contrary to others^24,25^, which found that females with SZ had higher CRP than male patients. Surprisingly, CRP showed a positive correlation with BMI in female subjects only, with previous studies observing a markedly stronger association between CRP and obesity in women than in men^26^.

It is noteworthy that sex hormones may play a role in modulating the relationship between inflammation and cognition^27^. Previous literature provided evidence of the capacity of estrogens to inhibit the inflammatory processes, both peripherally and in the brain^28^, and it is known that menopause and ovariectomy can generate a low grade of systemic inflammation^29^. Additionally, this neuroprotective action of estrogens seems to decrease over time, and it is plausible that with age a form of estrogen resistance is involved in the impaired ability of microglia to resolve inflammation^28^. These findings could be meaningful in mental disorders like schizophrenia, because women with schizophrenia tend to be hypoestrogenic and experience more severe symptoms, including cognitive impairment, in post-menopause than pre-menopause^30^. Notwithstanding, the underlying mechanisms of this association are not fully elucidated yet, and future studies are needed to clarify further the interaction of inflammation and sex hormones as a determining factor of cognitive performance.

Sex differences may also play a role in the response to different treatment strategies and could be relevant in the context of the mixed results obtained with both pharmacological and non-pharmacological strategies to ameliorate cognition in SZ^31^. An example of anti-inflammatory therapy is the tetracycline antibiotic minocycline, capable of crossing the blood–brain barrier and attenuating the inflammatory response, which showed a neuroprotective effect limited to males in both animal models of neurodegenerative diseases and patients^9^. Minocycline showed beneficial effects on negative and cognitive symptoms in SZ^3^, but we could not find any studies that compared cognitive outcomes as a function of sex in SZ. Another study found a sex-specific anti-inflammatory effect of aripiprazole, suggesting that aripiprazole may be protective against peripheral inflammation specifically in females with SZ^24^. With regards to non-pharmacological treatments, a recent work investigated the effects of a mindfulness intervention on older adults diagnosed with mild cognitive impairment and found a significant decrease in CRP levels in females only^32^. Although recent preclinical and clinical researches have paid attention to include female subjects, females are still underrepresented in many lines of investigation, including mental health disorders^33^. Thus, we agree with the current regulations, which require clinical trials to examine and report sex-related differences in the response to the investigational treatment.

The antipsychotic treatment represents another factor with potential impact on cognitive function^34,35^, and a higher chlorpromazine equivalent dose was a predictor of worse performance in the visual learning domain in males, but not in females. However, males were treated with a higher dose of antipsychotic drugs in our sample. A possible explanation for this might be that men also showed a more severe psychopathological presentation, with higher positive symptoms, and thus required greater doses of antipsychotics than females. These results are in agreement with existing data, which found that high-dose prescription was more frequent in men^36^. Additional studies are needed to elucidate further the interaction between antipsychotic treatment, inflammation, and cognition, and should therefore consider the potential differences in the dose between sexes. Moreover, the inclusion of antipsychotic doses in the analysis is important because of their possible intrinsic anti-inflammatory and immunomodulatory effects^3^.

However, our results need to be interpreted cautiously because of a series of limitations. Firstly, the cross-sectional design limits the possibility to draw conclusions about the association of CRP levels with cognitive decline over time. Longitudinal studies, especially ones with medium-long term follow-up, are needed to elucidate if there is a difference in cognitive decline in the subgroup with low-grade systemic inflammation at baseline, especially as regards the female sex. Secondly, peripheral CRP was the only marker of inflammation in our study, and we could not study its concentration in the cerebrospinal fluid. Also, we did not consider the hormonal status of tested female participants. Lastly, our study lacks a control group.

Strengths of our study include the careful clinical assessment of participants, particularly the use of internationally validated tests such as the MATRICS Consensus Battery and the assessment of negative symptoms with the CAINS. Moreover, our study examined the potential confounding or moderating effect of various factors, as described in the Methods section.

In conclusion, our results show a sex-specific modulation of the association between cognition and systemic inflammation, as measured by serum CRP levels, and suggest the existence of a subgroup of female patients who could benefit from anti-inflammatory treatments to ameliorate cognitive impairment. Therefore, clinicians should be aware of these results when designing treatment plans in their daily clinical practice, constituting yet another step toward implementation of a patient-centred approach in psychiatry in years to come.

## Supplementary information

Supplementary Information.

## Data Availability

We cannot make public the data generated during the present study because we do not have the consent of patients to publish their data. Interested researchers can contact the corresponding author for inquires or questions.
