# Supplementary material for: Cognitive impairment and C-reactive protein in clinically stable schizophrenia outpatients: a focus on sex differences
Source: Sci Rep. 2020 Sep 29;10:15963. doi: 10.1038/s41598-020-73043-x (PMC7524709; doi:10.1038/s41598-020-73043-x)
Supplement: Supplementary file 1 — Supplementary Information. [file 41598_2020_73043_MOESM1_ESM.pdf]

Cognitive impairment and C-reactive protein in clinically stable schizophrenia outpatients: a focus on sex differences.

Francesco Dal Santo, Leticia González-Blanco, Leticia García-Álvarez, Lorena de la Fuente-Tomás, Ángela Velasco, Clara María Álvarez-Vázquez, Clara Martínez-Cao, Pilar A. Sáiz, María Paz García-Portilla, Julio Bobes

Supplementary Information

Supplementary Table S1

Antipsychotic drug prescription in the sample (132 patients)

	N	%
Number of antipsychotic drugs prescribed per patient		
No antipsychotics	9	6.8
1 antipsychotic	77	58.3
2 antipsychotics	36	27.3
>2 antipsychotics	10	7.6
Antipsychotic drugs prescribed		
Amisulpiride	4	3.0
Aripiprazole	28	21.2
Clotiapine	2	1.5
Clozapine	7	5.3
Fluphenazine	2	1.5
Haloperidol	4	3.0
Levomepromazine	1	0.8
Olanzapine	36	27.3
Paliperidone	64	48.5
Quetiapine	14	10.6
Risperidone	18	13.6
Ziprasidone	1	0.8

Supplementary Table S2

Correlation between visual learning (MCCB T-score), and psychopathological variables [r (p)]

	Men (n=82)	Women (n=50)
PANSS Positive	-0.155 (0.166)	-0.198 (0.172)
PANSS Negative	**-0.488 (0.000)**	**-0.376 (0.008)**
PANSS GP	**-0.346 (0.001)**	**-0.320 (0.025)**
CAINS MAP	**-0.597 (0.000)**	**-0.448 (0.001)**
CAINS EXP	**-0.539 (0.000)**	**-0.340 (0.017)**
CDS	0.054 (0.627)	-0.166 (0.253)

CAINS: Clinical Assessment Interview for Negative Symptoms, -EXP: Expression subscale, -MAP: Motivation and Pleasure subscale, CDS: Calgary Depression Scale, CRP: C-Reactive Protein; MCCB: MATRICS Consensus Cognitive Battery, PANSS: Positive and Negative Syndrome Scale, -GP: General Psychopathology
